# Deficiency in Clonogenic Endometrial Mesenchymal Stem Cells in Obese Women with Reproductive Failure – a Pilot Study

**DOI:** 10.1371/journal.pone.0082582

**Published:** 2013-12-10

**Authors:** Keisuke Murakami, Harish Bhandari, Emma S. Lucas, Satoru Takeda, Caroline E. Gargett, Siobhan Quenby, Jan J. Brosens, Bee K. Tan

**Affiliations:** 1 Division of Reproductive Health, Clinical Science Research Laboratories, Warwick Medical School, University of Warwick, Coventry, United Kingdom; 2 Department of Obstetrics and Gynecology, Juntendo University Faculty of Medicine, Tokyo, Japan; 3 The Ritchie Centre, Monash Institute of Medical Research and Department of Obstetrics and Gynecology, Monash University, Clayton, Australia; 4 Department of Obstetrics and Gynecology, Birmingham Heartlands Hospital, Heart of England NHS Foundation Trust, Birmingham, United Kingdom; University of Warwick – Medical School, United Kingdom

## Abstract

**Objectives:**

The mechanisms of obesity associated reproductive complications remain poorly understood. Endometrial mesenchymal stem-cells are critical for cyclic renewal and uterine function. Recently, W5C5^+^ cells, with high clonogenicity, capable of producing endometrial stroma *in vivo*, have been described. We sought to investigate the abundance and cloning efficiency of W5C5^+^ and W5C5^−^ endometrial cells in relation to Body Mass Index, age and reproductive outcome.

**Design:**

W5C5^+^ and W5C5^−^ cells were purified from mid-luteal endometrial biopsies (n = 54) by magnetic bead separation and subjected to *in vitro* colony-forming assays.

**Results:**

First trimester pregnancy losses were significantly higher in obese subjects (n = 12) compared to overweight (n = 20) and subjects with normal Body Mass Index (n = 22) (*P*<0.05, *P*<0.01, respectively). W5C5^+^ cells (%) were significantly lower in obese subjects compared to subjects with normal Body Mass Index (*P*<0.05). W5C5^+^ cloning efficiency was significantly lower in obese subjects compared to overweight and subjects with normal Body Mass Index (*P*<0.05, respectively). W5C5^−^ cloning efficiency was significantly lower in obese subjects compared to subjects with normal Body Mass Index (*P*<0.05). Body Mass Index was significantly negatively correlated with W5C5^+^ cloning efficiency and W5C5^−^ cloning efficiency (*P*<0.01, respectively), and positively correlated with first trimester loss (*P*<0.01). We found no significant results with age (*P*>0.05).

**Conclusions:**

Our observations suggest that the regenerative capacity and plasticity of the endometrium of obese women is suboptimal, which in turn may account for the increased risk of reproductive complications associated with obesity.

## Introduction

The pandemic of obesity is one of today’s most blatantly visible global public health problem. Obesity is associated with adverse metabolic as well as reproductive outcomes such as miscarriage and infertility [1]–[3]. However, the mechanisms of obesity associated reproductive complications remain poorly understood. Consequently, there are no effective interventions that are available in the prevention and treatment of obesity associated reproductive disorders. We put forward a novel hypothesis that a suboptimal uterine environment in obese women at time of embryo implantation predisposes to reproductive failure.

A striking feature of the human endometrium, shared with only a handful of other mammalian species, is spontaneous decidualization of the stromal compartment during the mid-luteal phase of each cycle, a process also responsible for the menstrual shedding of the endometrium in the absence of pregnancy. Decidualization is characterised by transformation of endometrial stromal fibroblast into specialized secretory decidual cells, a process indispensable for embryo implantation [4]. Perturbations in decidualization can have negative effects on trophoblast invasion, placental development and, ultimately, maternal and fetal well-being [5]. An inevitable consequence of spontaneous decidualization followed by menstruation is the requirement for cyclic regeneration of the endometrium. The human endometrium exhibits remarkable regenerative capacity [6]. The endometrium is rich in mesenchymal stem-like cells (eMSCs), which are immuno-privileged compared to other types of stem-like cells, rendering them a promising resource for cell-based therapies [7]–[9]. They are a resident cell population, although there is some evidence of active recruitment of stem-like cells to hypoxic, proteolytic and inflammatory stimuli associated with cyclic menstruation or pregnancy [6], [10], [11]. This process of constant renewal bestows plasticity on the endometrium, enabling it to adapt to reproductive failure and a changing environment. Recently, Masuda et al. identified W5C5 as a novel single marker for purifying eMSCs that self-renew, have high clonogenicity, are multipotent (differentiate into adipogenic, osteogenic, chondrogenic and myogenic cell lineages) and are capable of producing endometrial stroma (mesodermal tissue) *in vivo* [12].

We sought to study the relationship between the abundance and cloning efficiency (CE) of W5C5^+^ and W5C5^−^ endometrial cells with body mass index (BMI), age and reproductive outcome.

## Materials and Methods

### Ethics

The study was approved by the NHS National Research Ethics - Hammersmith and Queen Charlotte’s & Chelsea Research Ethics Committee (1997/5065). The study has also been approved by the University Hospitals Coventry and Warwickshire Research and Development department, and research sponsorship for this study has been transferred from Imperial College, London to the University of Warwick, and written informed consent was obtained from all participants, in accordance with the guidelines in The Declaration of Helsinki 2000.

### Patient selection and endometrial sampling

A total of 54 Caucasian subjects were recruited consecutively from the Implantation Clinic, a dedicated research clinic at University Hospital Coventry and Warwickshire for women with recurrent pregnancy loss or recurrent *in vitro* fertilization treatment failure. Weight and height measurements were performed and BMI was calculated in all subjects. The World Health Organization classification of BMI, normal BMI (< 25), overweight (BMI: 25.0–29.9) and obese (BMI ≥ 30) was used. All endometrial biopsies were timed and histologically dated between 7 to 10 days after the pre-ovulatory luteinizing hormone surge. Samples were obtained using a Wallach Endocell^TM^ sampler (Wallach, Trumbull, USA) under ultrasound guidance, starting from the uterine fundus and moving downwards to the internal cervical ostium.

### Preparation of single cell suspensions of human endometrial stromal cells

Endometrial samples were collected as described above and single cell suspensions of human endometrial stromal cells (HESCs) were isolated using a protocol that was a modification of the method described [12.13]. Briefly, samples were washed in DMEM/F-12 medium (Invitrogen, Paisley, UK), finely minced and enzymatically digested with collagenase (0.5 mg/ml) (Sigma-Aldrich, Gillingham, UK) and deoxyribonuclease type I (0.1 mg/ml) (Roche, Burgess Hill, UK) for 1 hour at 37°C. The dissociated cells were filtered through a sterile 40 um cell strainer (Fisher Scientific, Loughborough, UK). Most of the stromal cells and blood cells, present as a single cell suspension, passed through the cell strainer, whereas the undigested fragments, mostly comprising of glandular clumps, were retained on the strainer. Stromal single cell suspensions were layered over Ficoll-Paque PLUS (GE Healthcare, Little Chalfont, UK) and centrifuged to remove erythrocytes. The medium/Ficoll-Paque PLUS interface, mainly containing stromal cells, was carefully aspirated, washed with DMEM/F-12 medium, and then subjected to magnetic bead separation.

### Magnetic beads separation

W5C5^+^ cells are perivascular in their location and are evenly distributed throughout the endometrium [12]. Magnetic bead separation was performed according to the manufacture’s instruction (Miltenyi Biotec, Bisley, UK) on a Wallach Endocell^TM^ core of approximately 1.5 cm from all study subjects. Briefly, equal amounts of freshly isolated endometrial stromal cell suspensions (up to 1×10^6^ cells/100 µl of 0.5% BSA in PBS: Magnetic Bead Buffer) were incubated with phycoerythrin (PE) conjugated Anti-W5C5 antibody (5 µl/1×10^6^ cells) (BioLegend, London, UK) on ice for 20 minutes. Then cell suspensions (up to 1×10^7^ cells/80 µl of Magnetic Bead Buffer) were incubated with Anti-PE MicroBeads (20 µl/1×10^7^ cells) on ice for 20 minutes. Cell suspensions (up to 1×10^8^ cells/500 ul of Magnetic Bead Buffer) were applied onto the MS columns in a magnetic field followed by washing with 500 µl of Magnetic Bead Buffer three times. The columns were removed from the magnetic field and W5C5^+^ cells were flushed out by firmly pushing the plunger with 1 ml of Magnetic Bead Buffer. Cell counts were performed after magnetic bead separation and the percentages of W5C5^+^ cells and W5C5^−^ cells were calculated.

### *In vitro* colony-forming assay

Freshly isolated W5C5^+^ and W5C5^−^ cells were seeded at a clonal density of 50 cells/cm^2^ (to ensure equal loading onto fibronectin-coated 60 mm culture dishes and cultured in growth medium: DMEM/F12 containing 10% dextran-coated charcoal-treated fetal bovine serum (DCC-FBS), 1% L-glutamine (Invitrogen, Paisley, UK), 1% antibiotic-antimycotic solution (Invitrogen, Paisley, UK), insulin (2 µg/ml) (Sigma-Aldrich, Gillingham, UK), estradiol (1 nM) (Sigma-Aldrich, Gillingham, UK) and basic fibroblast growth factor (10 ng/ml) (Merck Millipore, Watford, UK). The first medium change was after the first 7 days. Subsequently, media was changed every 3-4 days. Colonies were monitored microscopically to ensure that they were derived from single cells. Cultures were terminated at 15 days and stained with hemotoxylin. Clusters of ≥ 50 cells were counted and the CE was determined from the formula: CE (%)  =  (number of colonies/number of cells seeded) × 100.

### Statistical Analysis

Data were checked for normal distribution using histograms and the Kolmogorov-Smirnoff test. Data were analysed by Unpaired t test or ANOVA (post hoc analysis: Tukey’s test), depending on the number of groups compared. Data are means ± SEM. Pearson correlation was used for calculation of associations between variables. All statistical analyses were performed using GraphPad Prism 6 (GraphPad Software, Inc., La Jolla, USA) and SPSS version 21.0 (SPSS, Inc., Chicago, USA). *P*<0.05 was considered significant.

## Results

The demographic details, W5C5^+^ cells (%), W5C5^−^ cells (%), W5C5^+^ CE and W5C5^−^ CE of all participating subjects (n = 54) are presented in Table 1.

**Table 1:** Demographics, W5C5^+^ cells (%), W5C5^−^ cells (%), W5C5^+^ CE and W5C5^−^ CE.

Variable	All Subjects	BMI < 25.0	BMI 25.0-29.9	BMI ≥ 30.0
Age (years)	35.2±0.7	34.5±1.0	36.2±1.2	34.7±1.7
BMI (kg/m^2^)	26.7±0.7	22.5±0.3	26.6±0.4	34.5±1.5
Live Births	0.6±0.2	0.4±0.2	0.6±0.2	0.9±0.5
First Trimester Loss	3.3±0.4	1.9±0.4	3.2±0.5	5.9±0.1
W5C5^+^ cells (%)	7.0±0.5	8.1±0.9	7.0±0.8	4.9±0.9
W5C5^−^ cells (%)	93.0±0.5	91.9±0.9	93.0±0.8	95.1±0.9
Cloning Efficiency				
W5C5^+^ (%)	2.4±0.3	3.4±0.6	2.2±0.4	1.0±0.3
W5C5^−^ (%)	0.7±0.2	1.2±0.4	0.5±0.2	0.2±0.1

Data are mean ± SEM. CE  =  Cloning Efficiency.

All Subjects (n = 54); BMI < 25.0 (n = 22); BMI 25.0-29.9 (n = 20); BMI ≥ 30.0 (n = 12).

**Figure 1:**
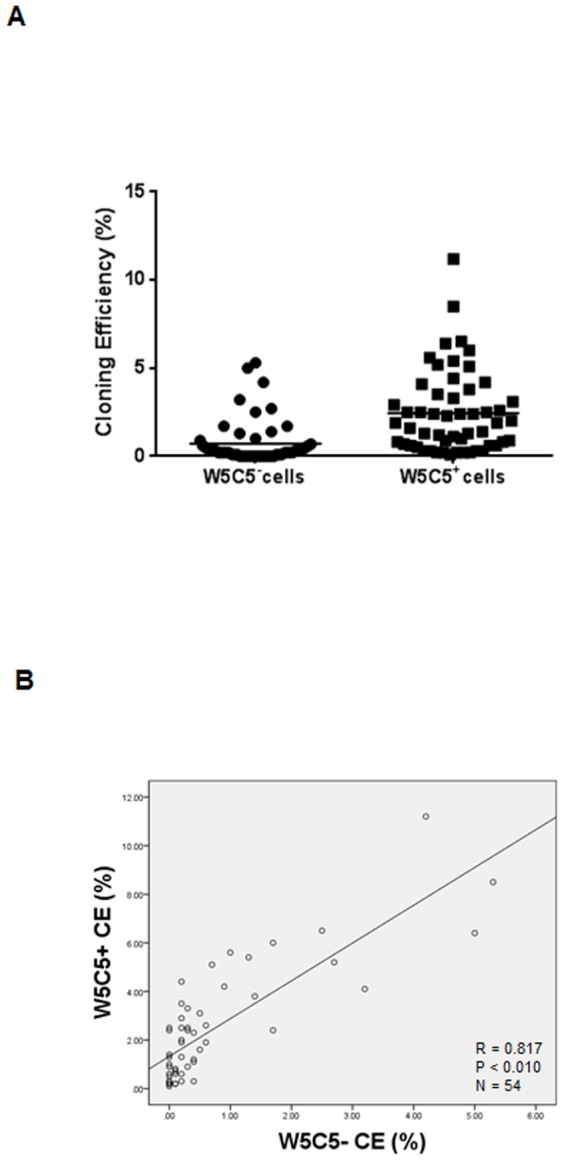
Cloning Efficiency (CE) of endometrial mesenchymal stem cells. (**A**) W5C5^+^ CE was significantly greater than W5C5^−^ CE. Data are means ± SEM. Group comparison by Unpaired t test. ****P<0.0001. (**B**) Relationship between W5C5^+^ CE and W5C5^−^ CE in all subjects (n = 54). Pearson correlation coefficient: (R = 0.817, *P*<0.01).

W5C5^+^ cells comprised 7.0±0.5% (n = 54) of stromal cells in the endometrium (Table 1). When compared pair-wise to W5C5^−^ CE, the W5C5^+^ population is on average 6-fold enriched in clonogenic cells (Figure 1A; ****P<0.0001). Furthermore, W5C5^+^ CE was significantly positively correlated with W5C5^−^ CE (Figure 1B; **P<0.01).

**Figure 2:**
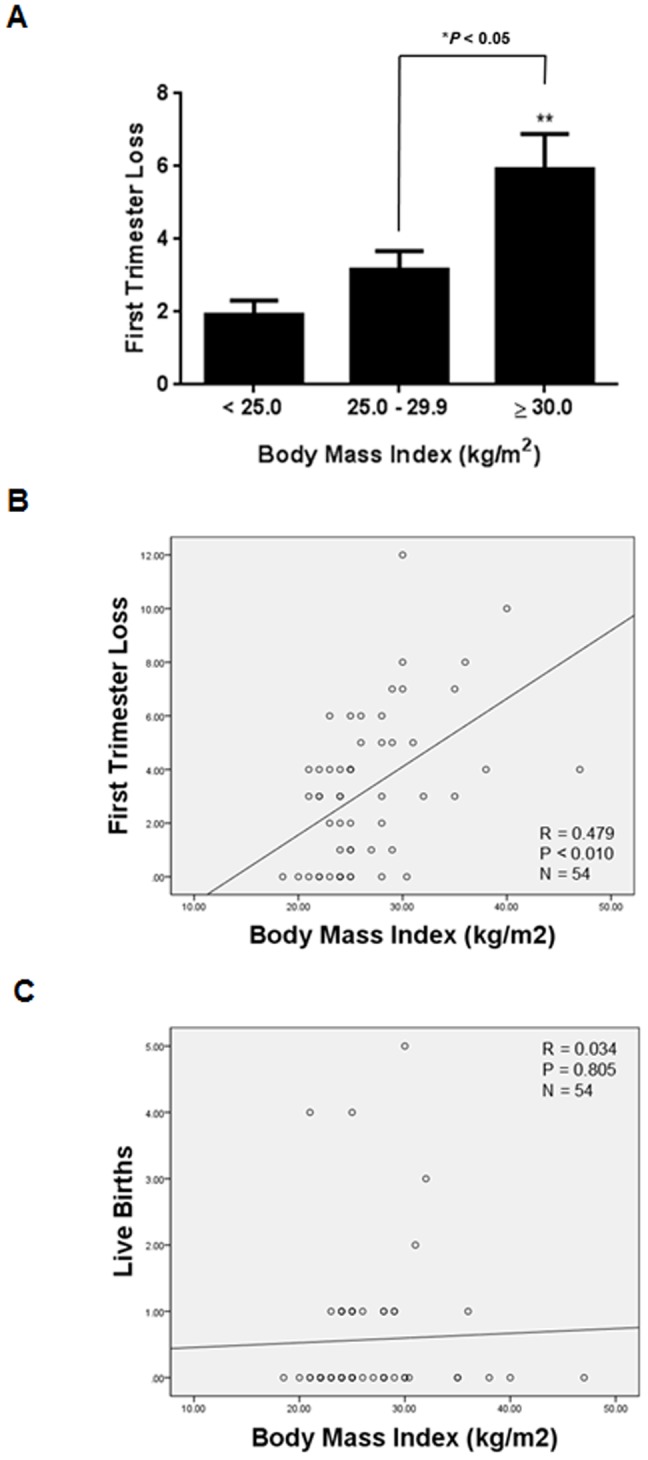
Body Mass Index and reproductive outcome. (**A**) First trimester pregnancy losses were significantly higher in obese subjects (n = 12) compared to overweight subjects (n = 20) and subjects with normal Body Mass Index (n = 22). First trimester losses in overweight subjects compared to subjects with normal Body Mass Index just failed to reach significance (*P* = 0.0565). Data are means ± SEM. Group comparison by ANOVA and post hoc Tukey’s test. *P<0.05, **P<0.01. (**B**) Relationship between first trimester pregnancy loss and Body Mass Index in all subjects. Pearson correlation coefficient: (R = 0.479, *P*<0.01). (**C**) Relationship between live births and Body Mass Index in all subjects. Pearson correlation coefficient: (R = 0.034, *P* = 0.805).

**Figure 3:**
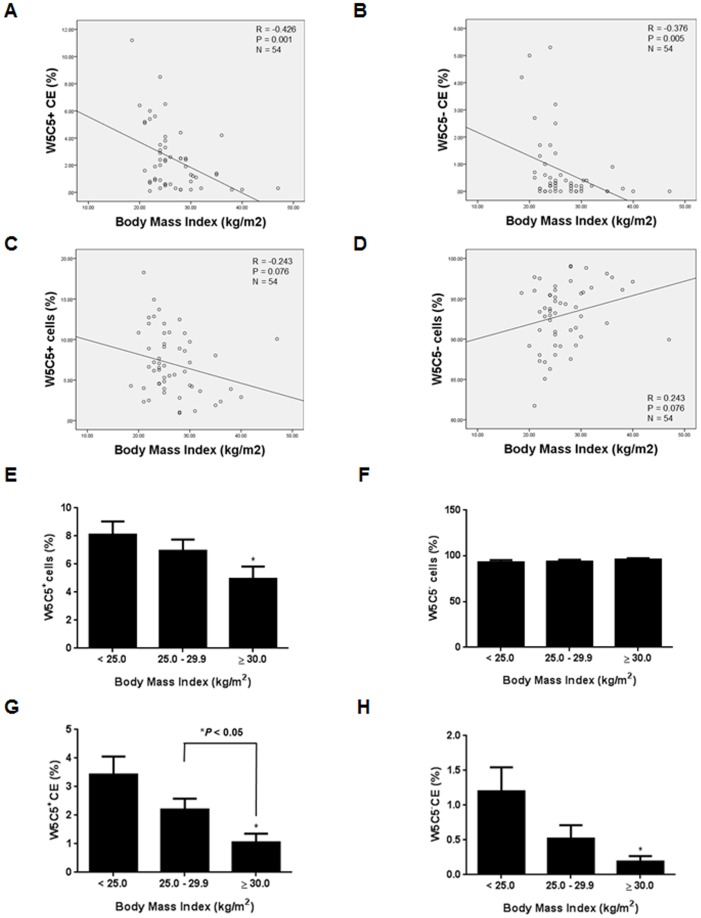
Body Mass Index and endometrial mesenchymal stem cells. (**A**) Relationship between W5C5^+^ CE and Body Mass Index in all subjects. Pearson correlation coefficient: (R = –0.426, *P* = 0.001). (**B**) Relationship between W5C5^−^ CE and Body Mass Index in all subjects. Pearson correlation coefficient: (R = –0.376, *P* = 0.005). (**C**) Relationship between W5C5^+^ cells (%) and Body Mass Index in all subjects (n = 54). Pearson correlation coefficient: (R = –0.243, *P* = 0.076). (**D**) Relationship between W5C5^−^ cells (%) and BMI in all subjects (n = 54). Pearson correlation coefficient: (R = 0.243, *P* = 0.076). (**E**) W5C5^+^ cells (%) were significantly lower in obese subjects (n = 12) compared to subjects with normal Body Mass Index (n = 22). Data are means ± SEM. Group comparison by ANOVA and post hoc Tukey’s test. *P<0.05. (**F**) W5C5^−^ cells (%) were not significantly different between obese, overweight (n = 20) and subjects with normal Body Mass Index. Data are means ± SEM. Group comparison by ANOVA and post hoc Tukey’s test. *P*>0.05. (**G**) W5C5^+^ CE was significantly lower in obese subjects compared to overweight subjects and subjects with normal Body Mass Index. Data are means ± SEM. Group comparison by ANOVA and post hoc Tukey’s test. *P<0.05. (**H**) W5C5^−^ CE was significantly lower in obese subjects compared to subjects with normal Body Mass Index. Data are means ± SEM. Group comparison by ANOVA and post hoc Tukey’s test. *P<0.05.

The number of previous first trimester pregnancy losses was significantly higher in obese subjects (n = 12) compared to overweight (n = 20) and subjects with normal BMI (n = 22) (Figure 2A; *P<0.05, **P<0.01, respectively). There was also a trend towards higher first trimester miscarriage rates in overweight subjects compared to subjects with normal BMI, although this failed to reach statistical significance (Figure 2A; *P* = 0.057). Moreover, BMI was correlated positively with first trimester loss (Figure 2B; **P<0.01) but not with live births (Figure 2C; *P*>0.05). This is in keeping with the report demonstrating that obese women are at an increased risk of recurrent pregnancy loss [14]. Also, BMI was correlated negatively with W5C5^+^ CE and W5C5^−^ CE (Figure 3A-B; **P<0.01).

**Figure 4:**
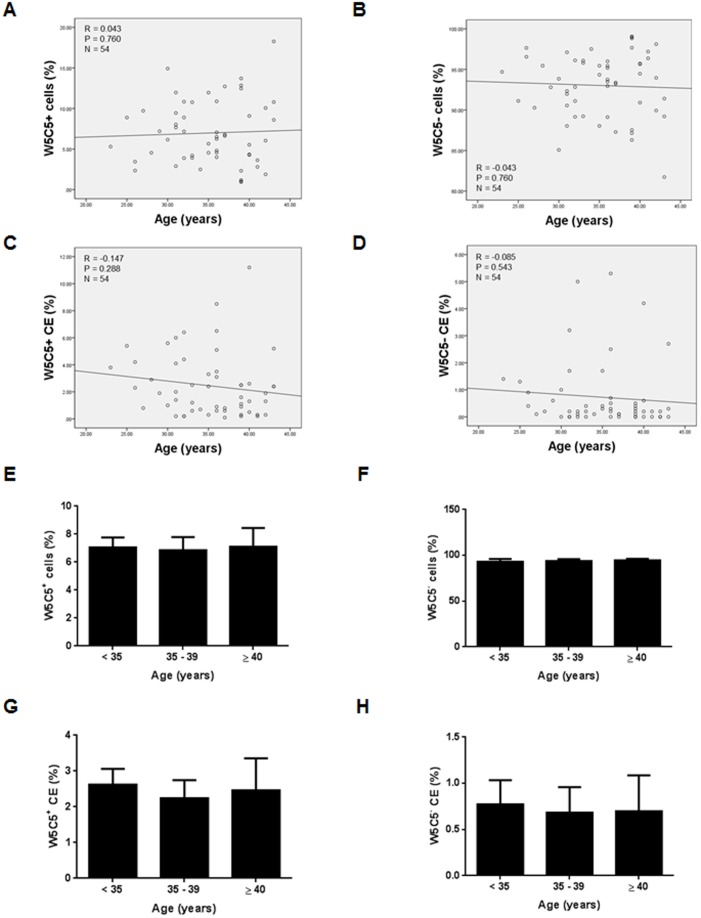
Age and endometrial mesenchymal stem cells. (**A**) Relationship between W5C5^+^ cells (%) W5C5^+^ CE and age in all subjects. Pearson correlation coefficient: (R = 0.043, *P* = 0.760). (**B**) Relationship between W5C5^−^ cells (%) and age in all subjects. Pearson correlation coefficient: (R = –0.043, *P* = 0.760). (**C**) Relationship between W5C5^+^ CE (%) and age in all subjects (n = 54). Pearson correlation coefficient: (R = –0.147, *P* = 0.288). (**D**) Relationship between W5C5^−^ CE (%) and age in all subjects (n = 54). Pearson correlation coefficient: (R = –0.085, *P* = 0.543). (**E**) W5C5^+^ cells (%) were not significantly different between subjects aged < 35 (n = 22), subjects aged 35–39 years (n = 20) and subjects aged ≥ 40 (n = 12). Data are means ± SEM. Group comparison by ANOVA and post hoc Tukey’s test. *P*>0.05. (**F**) W5C5^−^ cells (%) were not significantly different between subjects aged < 35, subjects aged 35–39 years and subjects aged ≥ 40. Data are means ± SEM. Group comparison by ANOVA and post hoc Tukey’s test. *P*>0.05. (**G**) W5C5^+^ CE was not significantly different between subjects aged < 35, subjects aged 35–39 years and subjects aged ≥ 40. Data are means ± SEM. Group comparison by ANOVA and post hoc Tukey’s test. *P*>0.05. (**H**) W5C5^−^ CE was not significantly different between subjects aged < 35, subjects aged 35–39 years and subjects aged ≥ 40. Data are means ± SEM. Group comparison by ANOVA and post hoc Tukey’s test. *P*>0.05.

BMI was not correlated significantly with the relative abundance (%) of either W5C5^+^ or W5C5^−^ cells (Figure 3C-D; *P*>0.05). Furthermore, age of the subjects was not correlated significantly with either the relative abundance (%) or the CE of either W5C5^+^ or W5C5^−^ cells (Figure 4A-D; *P*>0.05).

### Effects of BMI and age on W5C5^+^ cells (%), W5C5^−^ cells (%), W5C5^+^ CE (%) and W5C5^−^ CE (%)

The relative abundance (%) of W5C5^+^ cells was significantly lower in obese subjects compared to subjects with normal BMI (Figure 3E; *P<0.05). There were no significant differences in W5C5^−^ cells (%) between obese, overweight and subjects with normal BMI (Figure 3F; *P*>0.05).

However, W5C5^+^ CE was significantly lower in obese and overweight subjects compared to subjects with normal BMI (Figure 3G; *P<0.05, respectively). W5C5^−^ CE was also significantly lower in obese subjects (n = 12) compared to subjects with normal BMI (Figure 3H; *P<0.05).

There were no significant differences in W5C5^+^ cells (%), W5C5^−^ cells (%), W5C5^+^ CE or W5C5^−^ CE between subjects aged < 35 (n = 22), subjects aged 35–39 years (n = 20) and subjects aged ≥ 40 (n = 12), respectively (Figure 4E-H; *P*>0.05).

## Discussion

We report for the first time that obesity has a negative impact on the abundance of clonogenic eMSCs in the human endometrium of women with reproductive failure. Our findings suggest that the regenerative capacity and the plasticity of the endometrium in obese women is suboptimal, although it is important to bear in mind that other mechanisms, such as a chronic pro-inflammatory state, may also contribute to the increased risk of adverse reproductive outcomes in obese women [15]; further studies are needed to clarify this point.

The primary objective of this study was to investigate the abundance and CE of W5C5^+^ and W5C5^−^ endometrial cells and correlate this with BMI and reproductive outcome. However, given that the risk of miscarriages and infertility significantly increases at age 35 and age 40 [16], [17], we stratified our data into three age groups i.e. below 35 years, 35–39 years, and 40 years or older. There were no significant differences in the abundance or CE of W5C5^+^ and W5C5^−^ endometrial cells between the three age groups. Moreover, there were no significant correlations between the abundance and CE of W5C5^+^ and W5C5^−^ cells and age. These findings agree with the established notion that the age related decline in fecundity is associated with ovarian rather than uterine causes [18].

A limitation of our study may relate to the number of subjects studied. Nevertheless, we found a significant inverse correlation between BMI and CE of W5C5^+^ as well as W5C5^−^ cells. The clinical relevance of this finding is reinforced by the observation that the obese subjects had higher miscarriage rates as well as a deficiency in clonogenic eMSCs when compared to either overweight or normal women.

Our work has revealed important connections between W5C5^+^ endometrial cells with BMI and reproductive outcome, and would serve as a catalyst to promote further research into this exciting new area of stem cell research.

## Conclusions

Obesity is associated with a relative lack of clonogenic stromal cells in cycling endometrium. This may compromise the ability of the uterus to undergo intense tissue remodelling upon embryo implantation, thus increasing the risk of miscarriage and other pregnancy complications. Our data highlights a novel concept that pregnancy healthcare should be concentrated upon interventions that optimize the peri-implantation uterine environment in contrast to present research, which focuses on interventions after placental perfusion/pregnancy has already been established. This will potentially totally revolutionize the way we provide antenatal care to our patients. The precise mechanisms that govern the activity of endogenous eMSCs and the potential recruitment of clonogenic cells into cycling endometrium warrant further investigation.
